# Hyperglycemia screening based on survey data: an international instrument based on WHO STEPs dataset

**DOI:** 10.1186/s12902-022-01222-0

**Published:** 2022-12-14

**Authors:** Pooyan Moradifar, Hossein Amini, Mohammad Meskarpour Amiri

**Affiliations:** 1 Independent researcher, Tehran, Iran; 2 Health Management Research Center, Baqiyatallah University of Medical Sciences, Tehran, Iran

**Keywords:** Hyperglycemia, Logistic regression, Machine learning, Prediction models, Random forest, Screening, STEPs survey

## Abstract

**Background:**

Hyperglycemia is rising globally and its associated complications impose heavy health and economic burden on the countries. Developing effective survey-based screening tools for hyperglycemia using reliable surveillance data, such as the WHO STEPs surveys, would be of great importance in early detection and/or prevention of hyperglycemia, especially in low or middle-income regions.

**Methods:**

In this study, data from the nationwide 2016 STEPs study in Iran were used to identify socioeconomic, lifestyle, and metabolic factors associated with hyperglycemia. Furthermore, the ability of five commonly used machine learning algorithms (random forest; gradient boosting; support vector machine; logistic regression; artificial neural network) in the prediction of hyperglycemia on STEPs dataset were compared via tenfold cross validation in terms of specificity, sensitivity, and the area under the receiver operating characteristic curve.

**Results:**

A total of 17,705 individuals were included in this study, of those 29.624% (*n* = 5245) had (undiagnosed) hyperglycemia. Multivariate logistic regression analysis showed that older age (for the elderly group: OR = 5.096; for the middle-aged group: OR = 2.784), high BMI status (morbidly obese: OR = 3.465; obese: OR = 1.992), having hypertension (OR = 1.647), consuming fish more than twice per week (OR = 1.496), and abdominal obesity (OR = 1.464) were the five most important risk factors for hyperglycemia. Furthermore, all the five hyperglycemia prediction models achieved AUC around 0.70, and logistic regression (specificity = 70.22%; sensitivity = 70.2%) and random forest (specificity = 70.75%; sensitivity = 69.78%) had the optimal performance.

**Conclusions:**

This study shows that it is possible to develop survey-based screening tools for early detection of hyperglycemia using data from nationwide surveys, such as WHO STEPs surveys, and machine learning techniques, such as random forest and logistic regression, without using blood tests. Such screening tools can potentially improve hyperglycemia control, especially in low or middle-income countries.

## Background

Hyperglycemia is a metabolic disorder characterized by a high level of blood glucose, which in the intermediate stages appears in disguise of prediabetes—i.e. presence of impaired fasting blood glucose (IFG) and/or impaired glucose tolerance (IGT), or high glycated hemoglobin (HbA1c)—and could develop to diabetes mellitus (DM) if untreated [1]. Hyperglycemia is rising globally. Recent estimations show that 1 in 11 adults aged 20–79 have diabetes (463 million), and the global prevalence of IGT is estimated to be 7.5% (374 million) in 2019, accounting for 1 in 13 adults, and is projected to reach 8.0% (454 million) by 2030 and 8.6% (548 million) by 2045 [1, 2].

It is well-known that hyperglycemia is a precursor to a wide spectrum of non-communicable diseases (NCDs) such as DM, (cardio)vascular complications, diabetic retinopathy, and chronic kidney disease [1, 3]. These health complications in conjunction with the economic burden they impose on the countries necessitate serious measures for early detection and/or prevention of hyperglycemia [4]. To help better healthcare policymaking in this regard and to provide the information necessary for prevention programs, the STEPwise approach to surveillance (STEPs instrument) was proposed by the World Health Organization (WHO) as a standard and sustainable framework for collecting national data on risk factors of NCDs such as hyperglycemia [5, 6].

Several studies have documented an increasingly high prevalence of hyperglycemia in Iran. A recent study has reported the prevalence of prediabetes and diabetes 25.8% and 16.1% respectively among adults aged 20–69 years living in Yazd Province, Iran [7]. Another similar study has reported prevalence of prediabetes and diabetes 30.8% and 15.3% respectively among Iranian adults aged 20–65 living in Khuzestan Province, Iran, between 2016 and 2018 [8]. National surveys, conducted based on WHO STEPs instrument between 2005 and 2011, estimate 1 in 4 Iranian adults suffers from clinically significant abnormalities in glucose metabolism, and there has been a 5% annual increase in diabetes prevalence from 2005 to 2011. It is estimated that nearly 9.2 million Iranians are likely to have diabetes by 2030, and direct and indirect costs of diabetes alone will nearly triple from 2009, surpassing $9 billion in 2030 [4]. In developing countries like Iran, economic and financial restrictions to fully conduct national prevention programs, insufficient insurance coverage for laboratory and advanced technology diagnostics, and low access to standard diagnostic facilities in rural healthcare centers are amongst the main obstacles in controlling hyperglycemia [4]. Thus, developing simple and inexpensive screening tools for hyperglycemia would be of great importance.

Over the past few years, applications of machine learning (ML) in the screening and/or diagnosis of glycemic-based disordered have been well investigated. While most of the work in this area has focused on screening and/or diagnosis of diabetes [9, 10], there has also been some recent interest in shifting the focus towards hyperglycemia prediction as a precursor to diabetes. For instance, Choi et al. [11] and Deberneh et al. [12] have developed intelligence-based screening tools for hyperglycemia in the Korean population, demonstrating that ML methods can be useful in screening hyperglycemia. In another similar study, De Silva et al. [13] have employed ML techniques to identify predictors of hyperglycemia using a nationally representative sample of the US population.

In the present work, we aim to apply the five commonly used ML techniques (random forest; gradient boosting; support vector machine; logistic regression; artificial neural network) to the data from the nationwide 2016 STEPs study in Iran to identify non-invasive factors associated with hyperglycemia and develop predictive ML models using these factors. As a result, we propose ML predictive models for survey-based hyperglycemia screening, which can assist healthcare systems, especially in low or middle-income countries.

The structure of the paper is as follows: The mythology of the investigation is discussed in Methodology. The results are presented in Study population and discussed in Variable definitions and cutoff points. The conclusion of the paper is presented in Data analysis methods.

## Methodology

### Study population

Data from the latest Surveillance of Risk Factors of Non-communicable Disease (STEPs study) in Iran, conducted in 2016 based on WHO STEPs instrument, were used in this study [6]. The data comprise a sample of size *n* = 30,541 of Iranian population aged 18 and over, collected via cluster sampling from all provinces of Iran (except Qom province) in 2016; details of the Iran 2016 STEPs survey are discussed in [6, 14]. The final dataset used for analysis was formed from the original dataset according to the following inclusion–exclusion criteria: Participant for whom 1) Blood sugar measurements (fasting blood sugar (FBS) or HbA1c) were not provided, or 2) have already been diagnosed with hyperglycemia, or 3) have been taking medication for raised blood sugar, were excluded from the study. Pregnant women were also excluded from the study as their anthropometric measurements were not provided. The number of individuals who met the inclusion–exclusion criteria was 17,705, of which 12,460 (70.4%) had normal blood sugar, and 5245 (29.6%) had (undiagnosed) hyperglycemia. The baseline characteristics of the sample are presented in Table 1.

**Table 1:** Socioeconomic characteristics of the study participants

**Variables**	**Levels**	**Normal**	**Hyperglycemic**	**Total**	***p*-value**
Living area	Rural	4501 (70.53%)	1881 (29.47%)	6382 (36.05%)	0.7413
	Urban	7959 (70.29%)	3364 (29.71%)	11323 (63.95%)	
Gender	Male	5863 (69.89%)	2526 (30.11%)	8389 (47.38%)	0.1785
	Female	6597 (70.81%)	2719 (29.19%)	9316 (52.62%)	
Age group	Teenage	66 (84.62%)	12 (15.38%)	78 (0.44%)	<0.0001
	Young	7893 (85.27%)	1363 (14.73%)	9256 (52.28%)	
	Middle-aged	3035 (60.12%)	2013 (39.88%)	5048 (28.51%)	
	Elderly	1466 (44.12%)	1857 (55.88%)	3323 (18.77%)	
Income level	Very low	5478 (69.61%)	2391 (30.39%)	7869 (44.45%)	0.0149
	Low	6188 (71.33%)	2487 (28.67%)	8675 (49.0%)	
	Middle class	724 (67.79%)	344 (32.21%)	1068 (6.03%)	
	Upper middle class or higher	70 (75.27%)	23 (24.73%)	93 (0.53%)	
Education level	Illiterate	1516 (51.55%)	1425 (48.45%)	2941 (16.61%)	<0.0001
	Elementary school	3317 (67.5%)	1597 (32.5%)	4914 (27.75%)	
	High school	5049 (77.01%)	1507 (22.99%)	6556 (37.03%)	
	Academic	2578 (78.26%)	716 (21.74%)	3294 (18.6%)	
Marital status	Single	2299 (73.24%)	840 (26.76%)	3139 (17.73%)	0.0001
	Married	10161 (69.76%)	4405 (30.24%)	14566 (82.27%)	

### Variable definitions and cutoff points

Blood sugar level was determined based on FBS measurements in mg/dl, or HbA1c values. Hyperglycemia (the outcome variable) was defined as FBS ≥ 110 mg/dl according to WHO criterion [15], or HbA1c ≥ 5.7% according to the American Diabetes Association HbA1c criterion [3].

The 18 non-invasive principal input variables were selected based on the literature or their statistical significance in the univariate analysis. The principal input variables comprise socioeconomic variables, lifestyle variables (dietary pattern; smoking and alcohol consumption; physical activity), and metabolic risk factors (body-mass-index (BMI) status; abdominal obesity (AO); blood pressure (BP) status, hypertension history; having hypercholesterolemia (HC); history of HC).

Socioeconomic variables include: Living area (Urban/Rural); Gender (Male/Female); Age group (Teenager (age < 20); Young (age 20–45); Middle-aged (age 45–60); Elderly (age ≥ 60)); Income level (Very low; Low; Middle class; Upper-middle-class or higher); Education level (Illiterate; Elementary school; High school; Academic); Marital status (Married/Single); and Family type (Armed force households/Others).

Dietary pattern was determined using diet health level and fish consumption. Diet health level (poor diet; risky diet; healthy diet) was determined based on diet health score (DHS) in the range 0–12, calculated as the sum of the scores in the range 0–2 of six items (daily fruit/vegetable/dairy consumption; oil type used for cooking; salt consumption in each meal; monthly fast food consumption) according to IraPEN instructions [16]. Poor diet was defined as DHS < 5, risky diet was defined as DHS equal to 5 or 6, and healthy diet was defined as DHS ≥ 7. Fish consumption was determined based on weekly consumption in three categories (never/rarely; once or twice per week; more than twice per week).

Smoking status was defined based on daily cigarette smoking status at three levels: Non-smoker; former smoker; current smoker. Alcohol consumption was categorized as regular drinking (i.e. drinking at least 3 times per month) and non-regular drinking (including alcohol abstinence). Physical activity (PA) status was determined at four levels (low; moderate; high; intensive) based on weekly physical activity measured in MET-minutes using the Global Physical Activity Questionnaire [17]. Low/insufficient PA was defined as PA less than 600 MET-minutes per week; Moderate PA was defined as PA in the range 600–1500 MET-minutes per week; High PA was defined as 1500–3000 MET-minutes per week; Intensive PA was defined as PA at least 3000 MET-minutes per week [18].

BMI status was determined based on BMI values—calculated as weight measured in kilograms (kg) divided by the square of height measured in meters (m)—at five levels [19]: Underweight (BMI < 18.5); Normal BMI (18.5 ≤ BMI < 25); Overweight (25 ≤ BMI < 30); Obese (30 ≤ BMI < 40); Morbidly obese (BMI ≥ 40). Waist circumference (WC) and hip circumference were measured in cm. Abdominal obesity was defined based on WC or waist-to-hip ratio (WHR) criterion [19]: WC > 102 or WHR ≥ 90 for men, and WC > 88 or WHR ≥ 0.85 for women.

BP status was determined based on systolic blood pressure (SBP) and diastolic blood pressure (DBP), measured in mmHg, in three categories [20]: Normal BP (SBP < 120 and DBP < 80); Prehypertension (120 ≤ SBP < 140 or 80 ≤ DBP < 90); Hypertension (SBP ≥ 140 or DBP ≥ 90, or taking antihypertensive drugs). Pulse rate (PR) was defined as the number of heartbeats per minute. Pulse pressure (PP) was defined as ${\text{SBP}} - {\text{DBP}}$, and mean arterial blood pressure (MAP) was defined as ${{\left( {{\text{SBP}} + 2{\text{DBP}}} \right)} \mathord{\left/ {\vphantom {{\left( {{\text{SBP}} + 2{\text{DBP}}} \right)} 3}} \right. \kern-\nulldelimiterspace} 3}$. Hypertension history was defined as being diagnosed with high BP by a medical expert in the past. Having hypercholesterolemia (HC) was defined as being diagnosed with HC by a medical expert or taking medication for HC within the last year. Being diagnosed with HC in the past was defined as having an HC history.

### Data analysis methods

Statistical analysis of the data was performed by STATA v16.0 and statsmodels v0.13.1 module in Python. Categorical variables were presented by absolute frequency (*n*) and relative frequency (%), and were analyzed by Pearson's chi-squared test. Numerical variables were presented by mean ± standard deviation (SD) and were analyzed by Welch's ANOVA test. Multivariate logistic regression analysis was used to assess the simultaneous effect of the principal variables on the outcome variable (i.e. blood sugar status). In all the statistical tests a *p*-value less than 0.05 (*p* < 0.05) was regarded as statistically significant.

### Data cleaning and preprocessing

The dataset was thoroughly checked for the presence of inconsistencies or missing values. Inconsistences in the data were corrected according to the instructions in WHO STEPs Analysis Programs Documentation [21]. Missing values were imputed by the most frequent value (mode) for categorical variables, and mean value for numerical variables. Numerical variables were scaled via robust scaling technique, in which the values of each numerical variable are subtracted from their median and are divided by the interquartile range to reduce the effect of the outliers.

### Machine learning

Five commonly used machine learning algorithms—Random Forest (RF), Extreme Gradient Boosting (XGBoost), Support Vector Machine (SVM), Logistic Regression (LR), and Artificial Neural Network (ANN)—were developed to predict hyperglycemia. To optimize the performance of the models, hyperparameter optimization was done using the Bayesian optimization technique, as implemented in Python library scikit-optimize v0.8.1, with fivefold cross validation.

The Python machine learning library scikit-learn v0.24 was used to develop LR, RF, and SVM models. The XGBoost open-source software was employed to develop the XGBoost model. The ANN models were developed via Keras, the Python deep learning library, with 1 hidden layer (ANN1) and 2 hidden layers (ANN2) separately. The number of neurons in each hidden layer was determined via hyperparameter tuning while fitting the model. The ReLU function was used as the activation function for the input and hidden layers, and the sigmoid function was used as the activation function for the output layer. For training the ANN models, the categorical cross-entropy loss function was used, and the weights were optimized using Adam optimizer through 200 epochs.

For each of the five ML algorithms, four types (A, B, C, and D) of models were developed: Models of Type A are the baseline models built using the 18 principal input, ordinal encoded, categorical variables. Models of Type B were developed using dummy encoding of the principal input variables. Models of Type C and Type D are similar to models of type A and Type B respectively except that some additional numerical variables (age; the age of quitting smoking; log transform metabolic equivalent of task (MET); BMI; WC; WHR; pulse rate; pulse pressure; MAP) were included in the respective models.

### Model validation

The performance of the models was estimated via tenfold cross validation, and the mean ± SD value for performance metrics across the folds, as well as 95% confidence intervals (CI), were reported. The metrics used to measure the performance of the models include accuracy (Acc), specificity (SP), sensitivity (SN), and F1-score defined as follows in terms of the true positive (TP), false positive (FP), true negative (TN), and false negative (FN) cases [22]:

$$\begin{array}{c}\mathrm{Acc}=\frac{\mathrm{TN}+\mathrm{TP}}{\mathrm{TN}+\mathrm{TP}+\mathrm{FN}+\mathrm{FP}},\\ \begin{array}{c}\mathrm{SP}=\frac{\mathrm{TN}}{\mathrm{TN}+\mathrm{FP}},\\ \begin{array}{c}\mathrm{SN}=\frac{\mathrm{TP}}{\mathrm{TP}+\mathrm{FN}},\\ \mathrm{F}1=\frac{\mathrm{TP}}{\mathrm{TP}+\frac{1}{2}\left(\mathrm{FP}+\mathrm{FN}\right)}.\end{array}\end{array}\end{array}$$

The model performances were also compared in terms of the area under the receiver operating characteristic curve (AUC), which summarizes model performances in terms of sensitivity and specificity [22].

## Results

### Characteristics of the study participants

A total of 17,705 individuals with mean ± SD age 45.57 ± 15.263 were included in this study. The socioeconomic characteristics of the study participants are presented in Table 1. According to the table, almost half of the subjects were female (52.62%) and were young (52.28%), and most of the subjects were urban-dwellers (63.95%) and were married (82.27%).

The lifestyle and metabolic characteristics of the study participants are presented in Tables 2 and 3 respectively. According to the tables, around 70% of the subjects had unhealthy (poor or risky) diet, whereas a relatively low prevalence of current smoking (9.78%), regular alcohol drinking (0.83%), and low PA (13.75%) was observed among the subjects. Only 29.89% of the subjects had normal BMI, and 70.58% of the subjects were suffering from AO. More than half (66.59%) of the subjects had high BP, and 10.92% were suffering from HC.

**Table 2:** Lifestyle characteristics the study participants

Variables	Levels	Normal	Hyperglycemic	Total	*p*-value
Diet health level	Poor diet	1412 (72.93%)	524 (27.07%)	1936 (10.93%)	0.0302
	Risky diet	7885 (70.16%)	3354 (29.84%)	11,239 (63.48%)	
	Healthy diet	3163 (69.82%)	1367 (30.18%)	4530 (25.59%)	
Smoking status	Never smoked	10,835 (71.41%)	4338 (28.59%)	15,173 (85.7%)	< 0.0001
	Former smoker	480 (60.0%)	320 (40.0%)	800 (4.52%)	
	Current smoker	1145 (66.11%)	587 (33.89%)	1732 (9.78%)	
Regular drinking	No	12,342 (70.29%)	5216 (29.71%)	17,558 (99.17%)	0.0083
	Yes	118 (80.27%)	29 (19.73%)	147 (0.83%)	
PA status	Low PA	1697 (69.69%)	738 (30.31%)	2435 (13.75%)	0.0987
	Moderate PA	1645 (70.81%)	678 (29.19%)	2323 (13.12%)	
	High PA	6919 (69.9%)	2979 (30.1%)	9898 (55.91%)	
	Intensive PA	2199 (72.12%)	850 (27.88%)	3049 (17.22%)

**Table 3:** Metabolic characteristics of the study participants

**Variables**	**Levels**	**Normal**	**Hyperglycemic**	**Total**	***p*-value**
BMI status	Underweight	526 (77.13%)	156 (22.87%)	682 (3.85%)	<0.0001
	Normal BMI	4179 (78.97%)	1113 (21.03%)	5292 (29.89%)	
	Overweight	5160 (71.06%)	2101 (28.94%)	7261 (41.01%)	
	Obese	2485 (58.83%)	1739 (41.17%)	4224 (23.86%)	
	Morbidly obese	110 (44.72%)	136 (55.28%)	246 (1.39%)	
AO	No	4355 (83.61%)	854 (16.39%)	5209 (29.42%)	<0.0001
	Yes	8105 (64.86%)	4391 (35.14%)	12496 (70.58%)	
BP status	Normal BP	4911 (83.03%)	1004 (16.97%)	6316 (35.67%)	<0.0001
	Prehypertension	4562 (72.23%)	1754 (27.77%)	5474 (30.92%)	
	Hypertension	2987 (54.57%)	2487 (45.43%)	6316 (35.67%)	
HC	No	11393 (72.24%)	4379 (27.76%)	15772 (89.08%)	<0.0001
	Yes	1067 (55.2%)	866 (44.8%)	1933 (10.92%)	

The mean FBS of the subjects was 92.719 ± 20.804 mg/dl and the mean value for HbA1c was 5.485 ± 0.629. The prevalence of hyperglycemia in the sample was 29.624% (*n* = 5245), and the prevalence of hyperglycemia among men (30.11%) was slightly higher than women (29.19%), although the difference was not statistically significant. The mean age of the participants was 45.57 ± 15.263, and the mean age of the hyperglycemic subjects (54.36 ± 14.83) was significantly (*p* < 0.0001) higher than the mean age of the non-hyperglycemic subjects (41.874 ± 13.865).

### Predictors of hyperglycemia

The result of the multivariate logistic regression analysis for identifying significant predictors of hyperglycemia is presented in Table 4. Among the socioeconomic factors, gender, age group, and education level were significantly associated with hyperglycemia. While male gender (OR = 1.101) and older age (for the elderly group: OR = 5.096; for the middle-aged group: OR = 2.784) were identified as risk factors for hyperglycemia, higher education level was identified as a protective factor against hyperglycemia (for academic level: OR = 0.767; for high school level: OR = 0.851).

**Table 4:** Multivariate logistic regression analysis: Significant predictors of hyperglycemia

Variables	Levels	OR^a^	SE^b^	*z*-static	*p*-value	95% CI for OR
Gender	Male	1.101	0.047	2.26	0.024	1.013–1.197
	Female	1				
Age group	Teenage	1.414	0.457	1.07	0.284	0.75–2.665
	Young	1				
	Middle-aged	2.784	0.128	22.25	< 0.0001	2.544–3.047
	Elderly	5.096	0.293	28.30	< 0.0001	4.552–5.704
Education level	Illiterate	1				
	Elementary school	0.854	0.048	-2.82	0.005	0.765–0.953
	High school	0.851	0.053	-2.59	0.01	0.752–0.961
	Academic	0.767	0.054	-3.74	< 0.0001	0.667–0.881
Fish consumption	Never/Rarely	1				
	Once or twice	1.07	0.045	1.61	0.108	0.985–1.161
	More than twice	1.496	0.154	3.93	< 0.0001	1.224–1.83
Smoking status	Never smoked	1				
	Former smoker	1.171	0.098	1.87	0.061	0.993–1.38
	Current smoker	1.306	0.083	4.19	< 0.0001	1.153–1.48
PA status	Low PA	1				
	Moderate PA	0.908	0.063	-1.38	0.166	0.791–1.041
	High PA	0.957	0.052	-0.81	0.419	0.86–1.065
	Intensive PA	0.871	0.058	-2.07	0.038	0.765–0.993
BMI status	Underweight	1.27	.136	2.23	0.026	1.03–1.568
	Normal BMI	1				
	Overweight	1.268	0.062	4.86	< 0.0001	1.152–1.396
	Obese	1.992	0.111	12.35	< 0.0001	1.785–2.222
	Morbidly obese	3.465	0.502	8.57	< 0.0001	2.608–4.603
AO	No	1				
	Yes	1.464	0.073	7.68	< 0.0001	1.329–1.614
BP status	Normal BP	1				
	Prehypertension	1.367	0.066	6.43	< 0.0001	1.243–1.503
	Hypertension	1.647	0.084	9.76	< 0.0001	1.49–1.821
HC	No	1				
	Yes	1.27	0.069	4.39	< 0.0001	1.141–1.413

Multivariate logistic regression analysis adjusted for: Living area; Income level; Marital status; Family type; Diet health level; Regular alcohol drinking; Hypertension history; HC history

^a^*OR* Odds ratio, ^b^*SE* Standard error

Among the lifestyle factors, consuming fish more than twice per week (OR = 1.496), and current smoking (OR = 1.306) were identified as significant risk factors for hyperglycemia, whereas intensive PA was found to be a protective factor against hyperglycemia (OR = 0.038). As for metabolic factors, the results indicate that having abnormal BMI and high BP are significantly associated with increased odds of having hyperglycemia. It was also found that subjects with abdominal obesity (OR = 1.464) and HC (OR = 1.27) are more likely to have hyperglycemia.

### Model performances

**Fig. 1:**
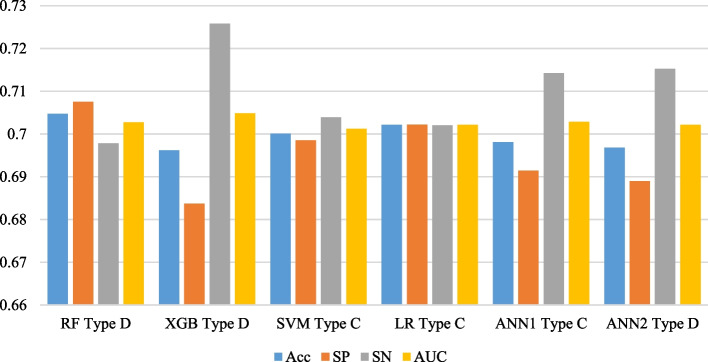
Performance of the optimal models

Five commonly used ML algorithms (RF; XGB; SVM; LR; ANN) were used for the prediction of hyperglycemia, and for each algorithm, four types of models (A, B, C, and D) were developed depending on the method of encoding used for categorical variables and inclusion of the numerical variables in the models. Among the four types of models developed for each ML algorithm, the RF Type D, XGB Type D, SVM Type C, LR Type C, ANN1 Type C, and ANN2 Type D were selected as the optimal models in terms of AUC, SN, and SP. The performance of the optimal models is presented in Table 5 and is compared in Fig. 1. The highest accuracy and specificity were achieved by RF Type D (Acc = 70.47%; SP = 70.75%) and LR Type C (Acc = 70.21%; SP = 70.22%) respectively. The highest sensitivity was achieved by XGB Type C (SN = 72.58%) and ANN2 Type D (SN = 71.52%) respectively. The AUC of all models was around 0.7, but the XGB Type D (0.7048) and RF Type C (0.7027) achieved the highest AUC respectively. Although all the models had similar performance in terms of AUC, the RF Type D and LR Type C were selected as the optimal models for predicting hyperglycemia as they had the optimal balanced performance in terms of AUC, specificity, and sensitivity.

**Table 5:** Performance of the optimal models via tenfold cross validation

**Models**		**Accuracy**	**Specificity**	**Sensitivity**	**AUC**	**F1-Score**
RF Type D	Mean	0.7047	0.7075	0.6978	0.7027	0.5835
	95% CI	(0.69, 0.72)	(0.69, 0.72)	(0.68, 0.71)	(0.69, 0.71)	(0.57, 0.6)
	SD	0.0178	0.0207	0.024	0.0178	0.021
XGB Type D	Mean	0.6962	0.6837	0.7258	0.7048	0.5861
	95% CI	(0.69, 0.71)	(0.67, 0.69)	(0.71, 0.74)	(0.69, 0.72)	(0.57, 0.6)
	SD	0.0177	0.0168	0.0233	0.0189	0.0215
SVM Type C	Mean	0.7001	0.6985	0.7039	0.7012	0.5818
	95% CI	(0.69, 0.71)	(0.69, 0.71)	(0.69, 0.72)	(0.69, 0.71)	(0.57, 0.59)
	SD	0.0151	0.0161	0.0219	0.0159	0.0185
LR Type C	Mean	0.7021	0.7022	0.702	0.7021	0.5827
	95% CI	(0.69, 0.71)	(0.69, 0.71)	(0.69, 0.72)	(0.69, 0.71)	(0.57, 0.59)
	SD	0.0143	0.013	0.0241	0.0164	0.0192
ANN1 Type C	Mean	0.6981	0.6914	0.7142	0.7028	0.5837
	95% CI	(0.69, 0.71)	(0.68, 0.7)	(0.7, 0.73)	(0.69, 0.71)	(0.57, 0.6)
	SD	0.016	0.0171	0.0276	0.0178	0.0207
ANN2 Type D	Mean	0.6968	0.689	0.7152	0.7021	0.583
	95% CI	(0.69, 0.71)	(0.68, 0.7)	(0.71, 0.73)	(0.69, 0.71)	(0.57, 0.59)
	SD	0.0157	0.0192	0.0163	0.0144	0.0166

ANN1 = ANN model with 1 hidden layer; ANN2 = ANN model with 2 hidden layers

## Discussion

In this study, the data of 17,705 records from the nationwide 2016 STEPs study in Iran were used for identifying socioeconomic, lifestyle, and metabolic factors associated with hyperglycemia. Furthermore, prediction models for the diagnosis of hyperglycemia were developed using five ML algorithms, and their performance was compared using tenfold cross validation in terms of accuracy, specificity, sensitivity, and AUC.

### Factors associated with hyperglycemia

Multivariate logistic regression analysis showed that socioeconomic factors (gender; age group; education level), lifestyle factors (weekly fish consumption; daily smoking), and metabolic factors (BMI status; abdominal obesity; BP status; having HC) were significantly associated with hyperglycemia. The five most influential risk factors were older age (for the elderly group: OR = 5.096; for the middle-aged group: OR = 2.784), high BMI status (morbidly obese: OR = 3.465; obese: OR = 1.992), having hypertension (OR = 1.647), consuming fish more than twice per week (OR = 1.496), and abdominal obesity (OR = 1.464).

The direct association between age and risk of having hyperglycemia, as well as the significant role of metabolic risk factors, such as obesity, (pre)hypertension, and having HC, in having hyperglycemia is quite well-established in the literature [3, 8, 23, 24].

The results of the study showed that men are slightly more susceptible to hyperglycemia than women (OR = 1.101), which is in accordance with several previous studies in Iran [8, 25], and the South Korean population [24]. The observation that higher education is protective against hyperglycemia has also been confirmed by several other studies in Iran [8, 25], but in a study based on the South Korean population, no significant association between education level and hyperglycemia have been reported [24].

Univariate and multivariate analysis did not show a significant association between the living area and hyperglycemia. However, some studies in Iran and other countries have confirmed that diabetes is more prevalent in urban areas whereas prediabetes is more prevalent in rural areas [8]. Although a significant association (*p* = 0.0149) between income level and hyperglycemia was observed in the univariate analysis, the multivariate logistic regression analysis did not show a significant association in presence of other factors. This observation is consistent with [24] but inconsistent with some other studies which show a direct association between income level and blood glucose level [2]. The observation that the prevalence of hyperglycemia is significantly higher (*p* < 0.0001) among married (30.24%) than single (26.76%) subjects is consistent with some studies in Iran [26]. However, no significant association between marital status and hyperglycemia was found in the multivariate analysis in presence of other factors, which contrasts some reports which suggest being single is protective against hyperglycemia [25].

We did not find a significant association between diet health levels, determined using IraPEN guidelines, with hyperglycemia. However, frequent fish consumption (more than twice per week) was found to be a significant risk factor for hyperglycemia (OR = 1.496). Inconsistent with this observation, some studies have not reported a significant association between fish consumption and diabetes [27], and some studies have reported an inverse relationship between fish consumption and the risk of diabetes [28]. These inconsistencies can in part be explained by the differences in the quantity and type of fish consumed, and also the differences in cooking methods in different food cultures [27, 29].

Multivariate regression analysis showed that subjects with intensive weekly PA are less likely to have hyperglycemia compared to those with low PA (OR = 0.871). Several studies have also reported the benefits of physical activity in the prevention of hyperglycemia [3, 30].

The role of smoking as a significant risk factor for hyperglycemia has been confirmed by many previous studies [8, 24], and a similar result was obtained in the present study. As for alcohol consumption, although hyperglycemia was found to be significantly more prevalent (*p* = 0.0108) among regular alcohol drinkers, no significant association between alcohol consumption and hyperglycemia was found in multivariate logistic regression analysis in the presence of other factors, which is contrary to the findings of some similar studies [23, 24, 31]. This inconsistency may in part be explained by a different pattern of alcohol consumption in Iran due to cultural and religious considerations.

### Prediction models for hyperglycemia

Among the models developed using the five ML algorithms, the LR Type C (AUC = 0.7021; SP = 70.22%; SN = 70.20%) and RF Type D (AUC = 0.7027; SP = 70.75%; SN = 69.78%) had the optimal balanced performance in terms of AUC, sensitivity, and specificity.

Logistic regression has previously been proposed as an optimal model for the prediction of hyperglycemia in several similar studies. In a study based on data from Tehran Lipid and Glucose Study in Iran [32], the proposed LR model (AUC = 0.71; SP = 65.58%; SN = 71%) was reported to outperform the ANN model in the diagnosis of hyperglycemia, similar to the findings of the present study. In several studies based on the Finnish Diabetes Risk Score, the sensitivity of the proposed LR models, using various combinations of variables, in the diagnosis of prediabetes was reported in the range 60–84%, whereas their specificity was reported in the range 53–61% [33, 34]. In comparison, the proposed LR Type C model in the present study seems to have a more balanced performance in terms of both specificity and sensitivity.

Several similar studies have also proposed the RF algorithm as an optimal algorithm for the prediction of hyperglycemia. In a study based on a nationally representative sample of the US population [13], a model based on RF (AUC = 0.7001; SP = 59.22%; SN = 72.33%) was reported to outperform LR, SVM, ANN, and gradient boosting algorithms in the prediction of prediabetes. Another study based on data from the South Korean population [12], compared predictive power of RF, SVM, LR, and XGBoost for prediabetes, and reported a similar performance for all the models, with slightly better performance for SVM (Acc = 73%; SN = 74%) and RF (Acc = 73%; SN = 72%) models. In comparison, the proposed RF Type D model in the present study seems to have a similar predictive power without using biochemical variables (such as triglycerides, uric acid) unlike the two aforementioned models [12, 13].

Maeta et al. [35] in a study based on data from a single facility in Tokyo, Japan, compared the performance of XGBoost and LR algorithms in the prediction of prediabetes using various combinations of features, including blood sugar measurements, and concluded that the XGBoost algorithm (AUC = 0.90; SN = 40.4%; SP = 97.4%) outperforms LR algorithm. In comparison, the proposed XGBoost model in the present study has lower AUC (0.7048) and specificity (68.37%), but has achieved a higher sensitivity (72.58%), and seems to have a more balanced performance in terms of sensitivity and specificity without using blood sugar measurements as an input variable.

Models based on the SVM algorithm were reported to perform better than the LR model in the prediction of prediabetes in some studies. Choi et al. [11] in a study based on the South Korean population compared the predictive power of SVM, ANN, and LR in the prediction of prediabetes, and reported SVM (AUC = 0.731; SP = 65.3%; SN = 69.4%) as the optimal model. In comparison, the SVM Type C model presented in this study has a lower AUC (0.7012) but has better performance in terms of SP (69.85%) and SN (70.39%). In another study based on data of 6500 subjects from 2005–2009 STEPs study in the Hamadan province, Iran, Tapak et al. [36] reported that the SVM algorithm (AUC = 0.979; SP = 100%; SN = 82%) outperforms LR and ANN algorithms in the prediction of hyperglycemia.

Although many studies, in accordance with the findings of the present study, have reported a weaker performance of ANN models in the prediction of hyperglycemia [13, 32], in a study by Liu et al. [9] the ANN model was reported to outperform LR and decision tree algorithm in the prediction of diabetes.

### Limitations

The present study has two main limitations. Firstly, at the time of preparing this work data from Iran's 2021 STEPs study, as well as STEPs studies conducted before 2016, were not available from National Institute for Health Research, Iran. These additional data could have been used for external validation, as well as developing more robust models. Another limitation is concerning the performance of the prediction models. Although all the proposed models achieved a fair performance (AUC ≥ 0.70), further performance improvement is necessary. Several studies have confirmed that prediction of hyperglycemia is more difficult than diabetes alone [11, 12], and the main reason for this seems to be the class overlap problem [37], caused by the similarity between characteristics of healthy and patients with prediabetes [12]. Therefore, developing techniques to overcome the class overlap problem in the context of hyperglycemia prediction seems to be necessary for significant improvement in prediction models.

## Conclusion

This study shows that it is possible to develop survey-based screening tools for early detection of hyperglycemia using data from nationwide surveys, such as WHO STEPs surveys, and machine learning techniques, such as random forest and logistic regression, without using blood tests. Such screening tools can potentially improve hyperglycemia control, especially in low or middle-income countries.

## Data Availability

The data that support the findings of this study are available from the National Institute for Health Research of Iran (NIHR) but restrictions apply to the availability of these data, which were used under license for the current study, and so are not publicly available. However, the data are available from the corresponding author upon reasonable request and with permission of NIHR.

## Abbreviations

Acc: Accuracy
ANN: Artificial neural network
AO: Abdominal obesity
AUC: Area under the receiver operating characteristic curve
BP: Blood pressure
BMI: Body mass index
CI: Confidence interval
DM: Diabetes mellitus
FBS: Fasting blood sugar
HC: Hypercholesterolemia
LR: Logistic regression
OR: Odds ratio
PA: Physical activity
RF: Random forest
SD: Standard deviation
SN: Sensitivity
SP: Specificity
SVM: Support vector machine
XGBoost: Extreme gradient boosting
